# Role of low‐impact‐factor journals in conservation implementation

**DOI:** 10.1111/cobi.14391

**Published:** 2024-10-17

**Authors:** Jonathan J. Choi, Leo C. Gaskins, Joseph P. Morton, Julia A. Bingham, Ashley M. Blawas, Christine Hayes, Carmen Hoyt, Patrick N. Halpin, Brian Silliman

**Affiliations:** ^1^ Duke University Marine Lab, Nicholas School of the Environment Duke University Beaufort North Carolina USA; ^2^ Duke University Marine Geospatial Ecology Lab, Nicholas School of the Environment Duke University Durham North Carolina USA; ^3^ Department of Environmental Engineering Sciences, Center for Coastal Solutions University of Florida Gainesville Florida USA; ^4^ Coastal Resources Center & RI Sea Grant, Graduate School of Oceanography University of Rhode Island Narragansett Rhode Island USA; ^5^ NOAA Northeast Fisheries Science Center Narragansett Rhode Island USA; ^6^ Department of Oceans Stanford University, Hopkins Marine Station Pacific Grove California USA; ^7^ National Ocean Service NOAA Silver Spring Metro Center I Silver Spring Maryland USA

**Keywords:** conservation, Endangered Species Act, journal impact factor, law, conservación, factor de impacto de las revistas, ley, Ley de Especies en Peligro de Extinción, 濒危物种法》, 保护, 法律, 期刊影响因子

## Abstract

Academic review, promotion, and tenure processes place a premium on frequent publication in high‐impact factor (IF) journals. However, conservation often relies on species‐specific information that is unlikely to have the broad appeal needed for high‐IF journals. Instead, this information is often distributed in low‐IF, taxa‐ and region‐specific journals. This suggests a potential mismatch between the incentives for academic researchers and the scientific needs of conservation implementation. To explore this mismatch, we looked at federal implementation of the United States Endangered Species Act (ESA), which requires the use of the “best available science” to list a species as endangered or threatened and thus receive powerful legal protections. In assessing the relationship between academic sources of this “best available science” and ESA implementation, we looked at the 13,292 sources (e.g., academic journals, books, reports, regulations, personal communications, etc.) cited by the second Obama administration (2012–2016) across all ESA listings. We compared the IFs of all 785 journals that published peer‐reviewed papers cited in these listings against their citation frequency in ESA listings to determine whether a journal's IF varied in proportion with its contribution to federal conservation. Most of the peer‐reviewed academic articles referenced in ESA listings came from low‐IF or no‐IF journals that tended to focus on specific taxa or regions. Although we support continued attention to cutting‐edge, multidisciplinary science for its ability to chart new pathways and paradigms, our findings stress the need to value and fund the taxa‐ and region‐specific science that underpins actionable conservation laws.

## Abstract (es)

El papel de las revistas con factor de impacto bajo en la implementación de la conservación

**Resumen:**

Los procesos de revisión académica, promoción y titularidad otorgan primas a la publicación frecuente en revistas con factor de impacto (FI) alto. Sin embargo, la conservación depende mucho de la información específica por especie, que es poco probable que tenga el atractivo necesario para las revistas de FI alto. En lugar de esto, esta información se distribuye con frecuencia en revistas de FI bajo, específicas para la región y ciertos taxones. Esto sugiere una desigualdad potencial entre los incentivos para los investigadores académicos y las necesidades científicas de la implementación de la conservación. Exploramos esta desigualdad mediante la implementación federal de la Ley de Especies en Peligro de Extinción de Estados Unidos (ESA), la cual requiere que se use la “mejor ciencia disponible” para enlistar a una especie como en peligro o amenazada para que así reciba una protección legal sólida. Durante el análisis de la relación entre las fuentes académicas de esta “mejor ciencia disponible” y la implementación de la ESA, revisamos 13,292 fuentes (revistas académicas, libros, reportes, regulaciones, comunicación personal, etc.) citadas en la segunda administración del presidente Obama (2012 – 2016) en todos los listados de la ESA. Comparamos los FI de las 785 revistas que publicaron artículos revisados por pares y citados en estos listados con la frecuencia de citas en los listados de la ESA para determinar si el FI de una revista varía en proporción a su contribución para la conservación federal. La mayoría de estos artículos referenciados en los listados de la ESA provenían de revistas con FI bajo o sin FI que tendían a enfocarse en regiones o taxones específicos. Aunque apoyamos la atención continua para la ciencia multidisciplinaria e innovadora por su capacidad de generar nuevos caminos y paradigmas, nuestros hallazgos resaltan la necesidad de valorar y financiar la ciencia específica por taxones y regiones que apuntala las leyes procesables de conservación.

## 低影响因子期刊在实施保护中的作用

**【摘要】** 学术评审、晋升和终身教职评审都非常重视在高影响因子期刊上频繁发表文章。然而, 保护通常依赖于物种特异的信息, 而这些信息不太可能具有高影响因子期刊所要求的广泛吸引力。相反, 这些信息通常会在低影响因子、针对特定类群或地区的期刊上发表。这表明对学术研究人员的激励与实施保护的科学需求之间可能存在不匹配。为了探究这种不匹配, 我们分析了美国联邦政府对《濒危物种法》(Endangered Species Act, ESA)的实施情况, 该法案要求使用“现有最佳科学”将物种列入濒危或受威胁物种名录, 从而获得强有力的法律保护。本研究收集了奥巴马第二届政府(2012‐2016年)在确定所有ESA名录时引用的13292项材料来源(如学术期刊、书籍、报告、法规、个人通信等), 以评估这种“现有最佳科学”的学术来源与ESA实施之间的关系。我们比较了在这些名录中引用的所有发表同行评审论文的4836种期刊的影响因子与它们在ESA名录中被引用的频率, 以确定期刊的影响因子是否与其对联邦政府的保护贡献成正比。我们发现, ESA名录中引用的大多数同行评审学术论文都来自低影响因子或无影响因子的期刊, 这些期刊往往关注特定类群或地区。我们支持继续关注前沿、多学科的科学, 因为它们能够开辟新的道路并建立新范式, 但与此同时, 本研究的结果也强调了需要重视和资助特定类群和地区的科学, 它们是保护法律实践的基础。**【翻译:胡怡思;审校:聂永刚】**

## INTRODUCTION

Academic review, promotion, and tenure (RPT) processes have notoriously contributed to a culture of publish‐or‐perish, where the number of high‐quality publications is a primary determinant for success in academia (van Dalen & Henkens, 2012). Although the number of publications is easy to assess, the quality of research is much more difficult. Impact factor (IF), originally intended as a tool for helping librarians assess which journals have the widest readership, emerged as an easy but imperfect proxy for quality, with the so‐called better science appearing in journals with a high IF (Schimanski & Alperin, 2018). This shortcut is particularly pronounced when RPT committees are unfamiliar with the field that the candidate works in or are otherwise stretched thin (Schimanski & Alperin, 2018).

Though RPT practices vary considerably by institution and some may place more emphasis on other evidence of readership and uptake, IF nevertheless shapes the broader academic community's perceptions of meaningful and worthwhile science (Callaway, 2016; Larivière & Sugimoto, 2019; McKiernan et al., 2019; Niles et al., 2020; Schimanski & Alperin, 2018; Walker et al., 2010). The work of Niles et al. (2020) shows a particularly revealing disconnect: younger, nontenured faculty highly value publishing as much as possible in prestigious journals, whereas those serving on RPT committees place much less emphasis on quantity and prestige. Thus, researchers may be drawn to publish in journals with high IF because it is perceived as prestigious and helpful for RPT, even if the RPT committees themselves value the quality of the underlying research, regardless of where it is published (Niles et al., 2020; Schimanski & Alperin, 2018).

Though the emphasis on quantifiable metrics arguably reduced the importance of patronage and nepotism, its emphasis on publication has potentially led to unintended consequences. Researchers have identified increased incentives for committing fraud or publishing in predatory open access journals to meet RPT demands (Carafoli, 2015; van Dalen & Henkens, 2012). The primacy of the publish‐or‐perish paradigm in English‐speaking institutions (Alperin et al., 2019; McKiernan et al., 2019; Schimanski & Alperin, 2018) may generate additional barriers for non‐Western academics who cannot afford the publication and conference attendance fees required to establish traditional academic credentials (Amutuhaire, 2022; Carafoli, 2015; van Dalen & Henkens, 2012). Further, these pressures have discouraged participation in policy processes and public engagement (Alperin et al., 2019; Otten, 2015; van Dalen & Henkens, 2012).

However, public engagement is uniquely important for conservation science. Given the crisis‐oriented nature of conservation work, the discipline has long placed a premium on protecting biodiversity (Soulé, 1985). Yet, conservation efforts often require baseline, species‐by‐species monitoring data (Lindenmayer & Likens, 2010; Lovett et al., 2007), which are not necessarily data that lend themselves to publication in high‐IF journals. Though conservation‐relevant pieces appear in high‐IF journals, these journals typically aim to reach a broader audience and generally pick articles accordingly. Thus, they are less likely to distribute the species‐specific population estimates and trends used in protecting individual species or places.

One potential avenue for exploring the mismatch between IF and the science needed for conservation is to look directly at how academic research is incorporated into regulations protecting biodiversity. In the United States, the Endangered Species Act (ESA) is a powerful legal tool for protecting biodiversity as it prevents the federal government from taking actions, such as issuing permits or changing management plans, which would jeopardize the continued existence of a threatened or endangered species. However, these protections only apply if a species is listed under the ESA as threatened or endangered. This makes the listing stage politically fraught and vitally important for the fate of a particular species. Congress thus requires that the ESA listing process be based solely on the “best scientific and commercial data available” (Endangered Species Act (1973), codified as amended at 16 U.S.C. §1533[b][1][A]).

By comparing the science underpinning the ESA and the science published in high‐IF journals, we present a novel attempt to quantify the mismatch in incentives between academic RPT and the research needs for improving federal conservation specifically. Though we do not mean to imply that academia's primary purpose should be in the service of federal policy making, we sought to show the previously underrecognized importance of research directly supporting conservation implementation and the need to properly value this type of applied research in the academy.

## METHODS

The US federal government is required, by statute, to use the “best scientific and commercial data available” when making their ESA listing decisions (16 U.S.C. §1533[b][1][A]). This standard is often referred to as the best available science standard. Given both the efforts of federal employees and the lurking threat of litigation for the use of inadequate science, we believe that the sources the federal government cites in listing species under the ESA represent the best available science for the purposes of the act.

We explored where this best available science came from and how it matches IF by looking at the references cited by the second Obama administration (2012–2016) to support their listing actions. We focused on the second Obama administration because its 260 listed species dwarf subsequent administrations: the Trump administration listed 22 species, and the Biden administration has listed 58. We looked at each of the 61 different listing actions to find the citations used in the finalized regulations. If the works cited were unavailable, we used either the proposed regulation, a species assessment document, or, for one flowering plant, the regulation designating its critical habitat. We then classified each reference in the works cited by reference type, including academic journals, personal communication, reports (including citations to laws and regulations), books, or other miscellaneous references. Sources that were cited multiple times in different listing rules were listed multiple times in our database.

Then, per journal, we compared the journal's IF and the journal's importance in ESA listings. We began with IF, which was calculated per journal as (Garfield, 1994)

(1) $$\mathrm{IF}=\frac{\text{Citations in the current year to articles published by the journal in the previous 2 years}}{\text{Number of articles published by the journal in the previous 2 years}}.$$

To calculate an average IF for each journal, we took each article, recorded the IF at the year closest to publication in the Journal Citation Reports database, and then, by journal, averaged over all the articles (Clarivate, 2023). This weighted the journal's IF as it might have been interpreted by a reader closer to the time of publication. Because the online database is limited to data from 1997 onward, all references older than 1997 were imputed to 1997. If a journal changed names, we used the current name for the journal and the IF closest to the date of publication for the article.

This method of averaging IF across years is necessarily limited. The IF is already a summary statistic because it reports the number of times an average article is cited. That means that some highly cited papers will contribute more to a journal's IF than others. Further, since the IF used in our averaging is taken from the year of publication, older publications will generally have a lower IF because the number of citations has generally gone up with the advent of the internet and electronic research distribution (Ariza‐Guerrero & Blázquez, 2023). This is particularly true because our data set is from 2012 to 2016; journals may have changed their focus or target audience since then. Further, ESA listings could include references over 100 years old, long before the advent of IF (though only 337 of the 4836 citations to academic journals are older than 1975, the year IF was first published) (Larivière & Sugimoto, 2019). Despite these limitations, IF is nevertheless a quantifiable metric of academic readership that is used in some RPT processes and has a history that goes back far enough to cover many of the sources used in the ESA listings (McKiernan et al., 2019).

We then attempted to characterize each journal's importance in ESA listings (i.e., assign a score to journals that represented the impact of that journal on ESA listings [or an ESA listing IF]). To do this, we mirrored the calculation of IF. For each article, we found the number of articles published in the previous 2 years by the same journal and then, by journal, averaged across all articles. For articles published prior to 1998, we used the number of articles published in 1997 and 1998 (as opposed to 1996 and 1997) because the online database does not have values prior to 1997. By dividing the number of citations to the journal in the ESA listings by this averaged number of published articles, we calculated a rough ESA listing IF scaled to the research output of the journal:

(2) $$\text{ESA listing IF}=\frac{\text{Number of citations to the journal in the ESA listings}}{\begin{array}{c} \text{Average number of articles published by the journal in the 2 years}\\ \text{preceding the publication of each cited article} \end{array}}.$$

Thus, if a journal was cited 5 times in the ESA listings and if in the 2 years before the publication of these 5 articles the journal published on average 50 articles annually, the ESA listing IF would be 5/50 or 0.1. For those journals that did not appear in the Journal Citation Reports database, we used both the average IF and the average number of citable items for journals with an IF <1 instead because we assumed that the journal was not listed because its readership was too small to be assessed by Clarivate.

We used the tidyverse package in R for our analyses and visualizations (R Core Team, 2021; Wickham et al., 2019). Code and data are included in Appendices S1 and S2.

## RESULTS

**FIGURE 1:**
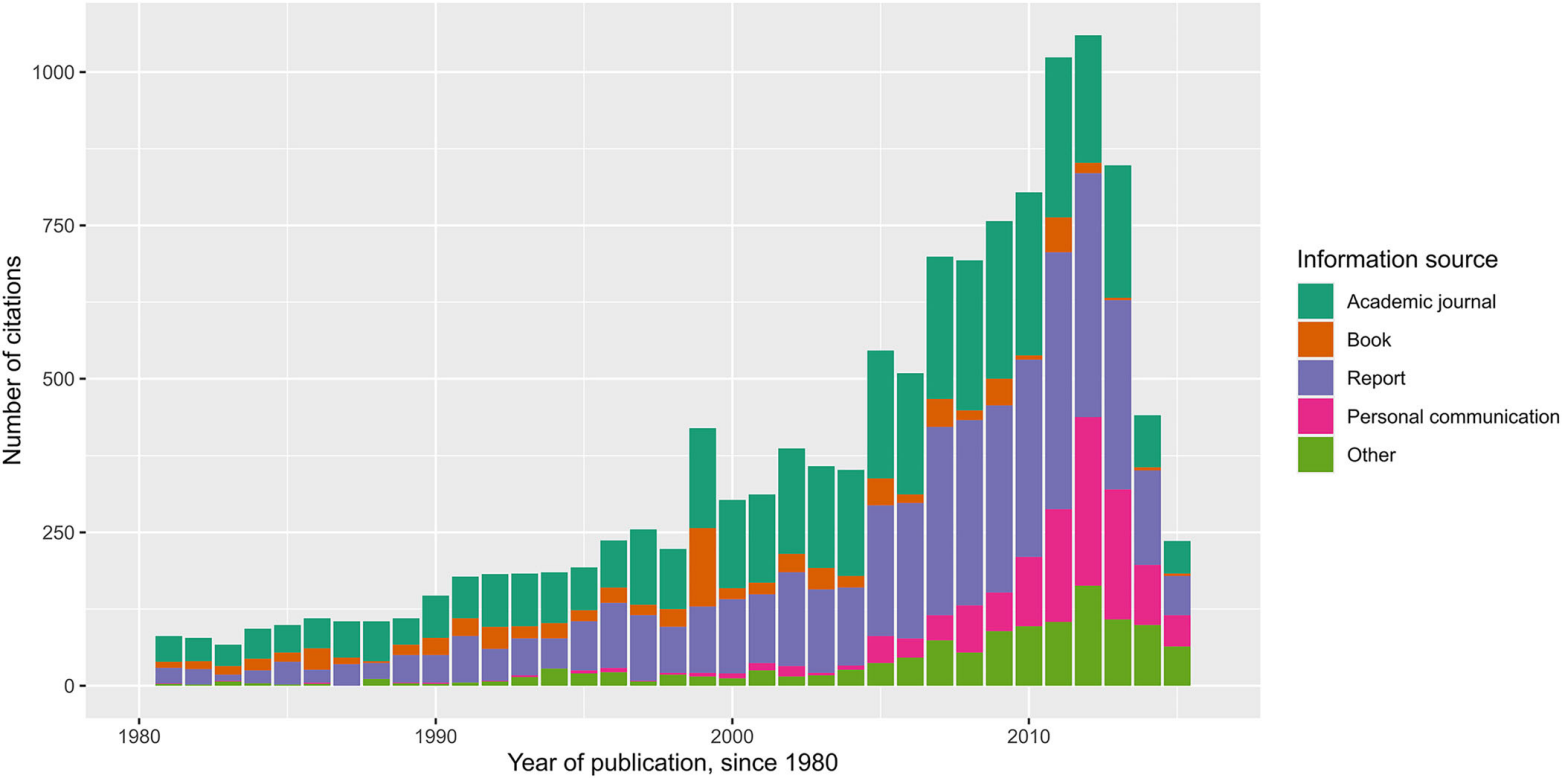
Types of sources cited in US Endangered Species Act listings from 2012 to 2016. Only items published since 1980 are included.

The second Obama administration cited 13,292 sources to support the listing of 260 species across 61 different regulatory actions. Many of the citations were to nonacademic sources, including personal communication and reports (Figure 1). Figure 1 shows the other cited sources that have been published since 1980. We excluded older dates for visualization purposes. Of the total data set, 4836 citations were to academic journals (∼36% of total citations) spread across 785 different journals.

**FIGURE 2:**
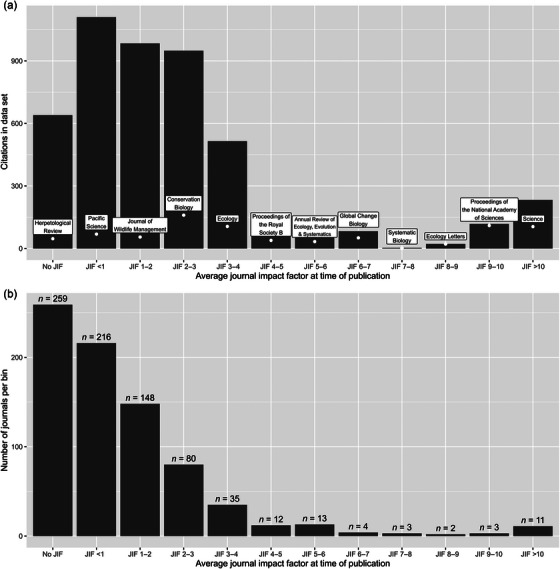
For journals cited by federal agencies to support US Endangered Species Act listings from 2012 to 2016, (a) number of citations per impact factor bin and number of citations from the most cited journal per bin and (b) number of journals that comprised each bin.

Many of the most frequently cited journals aligned with our expectations of prominent conservation journals, including *Conservation Biology*, *Proceedings of the National Academy of Sciences*, and *Ecology* (Table 1). However, IF alone did not predict the number of citations in our data set (Appendix S3). Rather, many citations came from numerous different journals with lower IFs (Figure 2). This mismatch was even more obvious when sorting based on ESA listing IF (Table 2). Table 2 shows the high number of region‐ or taxa‐specific journals that were cited far more often than their IFs would suggest.

**TABLE 1:** Twenty journals cited most frequently to support US Endangered Species Act listings from 2012 to 2016 and the average journal impact factor in the years the articles were published.

Journal	Citations in listings	Average impact factor
*Conservation Biology*	160	2.96
*Proceedings of the National Academy of Sciences*	110	9.87
*Ecology*	106	3.53
*Science*	105	28.43
*Coral Reefs*	92	2.81
*Biological Conservation*	88	2.47
*PLoS One*	73	3.84
*Pacific Science*	69	0.74
*Environmental Toxicology & Chemistry*	66	2.31
*BioScience*	64	3.10
*Ichthyology & Herpetology*	62	0.75
*Ecological Applications*	59	3.35
*Journal of Wildlife Management*	55	1.24
*Environmental Biology of Fishes*	53	0.81
*Freshwater Science*	53	2.05
*Rangeland Ecology & Management*	52	0.70
*Journal of Mammalogy*	52	1.21
*Global Change Biology*	51	6.48
*Marine Ecology Progress Series*	48	2.34
*Nature*	48	30.05

**TABLE 2:** Journals by US Endangered Species Act (ESA) listing impact factor, comparing number of citations in ESA listings with citable research items published by the journal.

Journal	Citations in listings	Average journal impact factor	Average no. citable items	ESA listing impact factor
*Pacific Science*	69	0.74	89.94	0.76
*Annual Review of Ecology, Evolution, & Systematics*	33	5.06	43.73	0.75
*Bulletin of the American Museum of Natural History*	5	1	7	0.71
*Coral Reefs*	92	2.80	161.77	0.57
*Freshwater Science*	53	2.05	100.51	0.53
*Conservation Biology*	160	2.96	306.55	0.52
*BioScience*	64	3.10	141.78	0.45
*Fisheries*	19	1.13	43.05	0.44
*American Malacological Bulletin*	9	0.48	24.22	0.37
*American Fern Journal*	12	0.61	35.08	0.34
*Southwestern Naturalist*	42	0.24	124.26	0.34
*Great Lakes Entomologist*	5	0.10	15	0.33
*Reviews in Fish Biology & Fisheries*	8	2.23	25.5	0.31
*Ecological Monographs*	15	5.72	50.27	0.30
*Ichthyology & Herpetology*	62	0.75	217.08	0.29
*Journal of the Lepidopterists Society*	18	0.52	64	0.28
*American Midland Naturalist*	46	0.54	165.15	0.28
*Herpetological Monographs*	3	1.13	11	0.27
*Rangeland Ecology & Management*	52	0.70	192.10	0.27
*Herpetological Review*	47	Not listed (0.62)^a^	184.04	0.26
*Nature* ^b^	48	30.05	1903.19	0.03
*Science*	105	28.43	1839.78	0.06
*Nature Climate Change*	16	14.94	171.25	0.09

*Note*: The table shows the number of citations in listing documents relative to citable items in the journal.

^a^ Journals without an impact factor were imputed to the average impact factor of journals with an impact factor from 0 to 1.

^b^ The last 3 journals had the highest journal impact factors.

## DISCUSSION

Our results suggest that academic RPT may undervalue taxa‐ and region‐specific science supporting conservation implementation because of its publication in low‐IF journals. The prevalence of low‐IF, taxa‐ and region‐specific natural history journals suggests that they play a vital role in attaching significant ESA protections (Table 2; Figure 2). Though our analysis is not sensitive enough to determine the content of the articles cited or what they were being cited for, we believe that the journals’ publication of natural history or species‐ and population‐level specific studies provides critical baseline data used in ESA listings. However, because these studies may not be of broader interest and thus are more difficult to place in high‐IF journals, academics may have less incentive to pursue this type of research. We believe that this may contribute to the general undervaluation of public‐facing aspects to conservation work. Though the ESA is only one model of conservation (and one that can always be improved [Hartl & Owley, 2021]), we believe that the pattern of undervaluation may extend beyond the United States.

Tables 1 and 2 show the large number of citations to taxa‐ and region‐specific journals. Journals such as *Coral Reefs*, *Pacific Science*, *Ichthyology & Herpetology*, and the *Journal of Mammalogy* were cited more often than *Nature*, the journal with the highest IF in the listings (Table 1). When accounting for research output through the ESA listing IF, taxa‐specific journals (e.g., *Coral Reefs*, *Freshwater Science*) and regional journals (e.g., *Pacific Science*, *Southwestern Naturalist*, *Great Lakes Entomologist*) were cited far more frequently than higher IF journals (Table 2; Figure 2). For instance, proportionally more of the research from the *American Fern Journal* was cited than research from *Nature* or *Science* (Table 2). This is perhaps an obvious finding, as the *American Fern Journal*’s audience will be much more concerned with reports of a rare fern than *Science*’s audience. However, this points to the unique ability of taxa‐ and region‐specific journals to answer the primary question of ESA listing: whether a species or population is “in danger of extinction throughout all or a significant portion of its range” or will be in the foreseeable future (16 U.S.C. § 1533[b][1][B]).

Indeed, our results suggest that academic research generally may not be contributing as much to ESA implementation as might be expected. Figure 1 shows that most citations are to nonacademic sources. Though some of these are nonscientific references (for instance, many citations classified as a report include citations to government regulations and laws that are used to establish the government's authority), we were surprised by the amount of personal communication that supported the listings. Anecdotally, this included phone calls to nonprofit organizations, other government researchers, and occasional academics.

Though there is undoubtedly variation in how academic departments prioritize conservation, ranging from the culture at a theory‐focused biology department to an application‐focused conservation and wildlife management department, we believe that the differences between IF and ESA listing IF and the number of citations to nonacademic sources suggest a potential misalignment in the priorities of academia and conservation implementation (Table 2; Figure 2). Generalizing broadly, academic RPT and its emphasis on IF encourage groundbreaking, multidisciplinary science that can provide insights into fundamental ecological phenomena. The resulting science enriches the field's understanding of bedrock principles in ecology, such as mutualism, competition, and predator–prey cycles, but fails to provide the baseline monitoring and basic natural history information about rare species that endangered species management requires. The academic publications supporting ESA listing lend themselves not to sweeping innovation in ecological theory but rather to tracking individual populations.

Our results also add evidence for the undervaluation of public‐facing aspects of academic research (Alperin et al., 2019). For example, maintaining and funding databases supporting conservation work can go underappreciated beyond the initial publication announcing the data set, potentially explaining the number of databases housed in nonacademic entities (e.g., the Seabird Tracking Database, MoveBank, and the Ocean Biodiversity Information System) (BirdLife International, 2023; Grassle & Stocks, 1999; Halpin et al., 2009; Kays et al., 2022). Programs focused on cultivating interest in natural history are often not the highest priority for top tier research universities but are immensely important to developing a constituency for conservation (McKeon et al., 2020; Tewksbury et al., 2014). A reconsideration of the academy's role in broader society might suggest a need for a deeper connection with natural history, ecological data collection, database maintenance, and federal research and management needs.

We believe that the undervaluation of academic science that contributes directly to conservation may extend beyond the United States, particularly where IF drives RPT. Though there are no global surveys of RPT in ecology departments, global surveys of demographers (van Dalen & Henkens, 2012) and early career scientists (Nicholas et al., 2017) show that perceived career benefits of publishing in high‐IF journals drive decisions about where to publish. Concurrently, species‐ and population‐specific natural history studies continue to drive international conservation, particularly when deciding to include species on conservation lists and when identifying sites of global conservation concern. Listing individual species based on population‐specific data remains a popular method for targeting conservation, both by national governments (e.g., Korean National Institute of Biological Resources, 2014; National System of Conservation Areas of Costa Rica, 2024) and by international bodies (CITES, 2024; CMS, 2020; IUCN, 2024). Similarly, site‐specific conservation efforts rely on species‐specific information. For example, BirdLife International works with local partners to identify important bird and biodiversity areas using individual site‐based surveys, tracking and tagging studies, and other field methods (Donald et al., 2019). This work is then incorporated into international treaty mechanisms (Davies et al., 2021; Waliczky et al., 2019). Yet, although academic scientists contributed to the research, it was the nonacademic institution that collated the data and led the direct engagement with policymaking bodies. Thus, given the international primacy of IF and the use of species‐ and population‐specific data for international conservation, we believe that the pattern revealed in our analysis of ESA listing data may hold true internationally.

Of course, we do not mean to say that academia should abandon innovation in favor of pure natural history, population surveys, or policy engagement. However, if the conservation community values protecting biodiversity, the value of baseline, natural‐history‐oriented research that feeds directly into conservation programs and reward scientists for their success in this area must be recognized. Insofar as it remains a goal of conservation to protect individual species from extinction, conservation science ought to seriously consider expanding the incentive structure created by current RPT processes to include incentives to pursue the basic, species‐ and population‐specific research that enables protection for threatened and endangered species, whether for ESA purposes or conservation more broadly.

Within academic departments, changing RPT criteria and a de‐emphasis of IF could provide breathing room for faculty, particular early career faculty, to engage the public (Nicholas et al., 2017). Efforts to raise the visibility of nonacademic uses for research have included efforts such as Altmetrics, which provide alternative measures for an article's reach, including the number of times research is shared on social media and saved in citation managers (Trueger et al., 2015). Though the method also includes citations to public policy documents, it does not necessarily encourage long‐term engagement with policy makers. Departments could encourage this public‐facing work by having faculty self‐identify the publications that they believe have the most societal importance as part of RPT or otherwise submit nonpublication evidence of their efforts to engage either policy makers or the public.

Beyond RPT, institutions can encourage public‐facing research. Models could be built around the cooperative extension model at US land grant institutions, where institutions receiving US federal funding are encouraged to meet the research needs of local communities (Ramussen, 2002). Hiring extension specialists in departments could help ensure that research with policy implications makes its way to policy makers. In teaching and research, incorporating natural history and teaching basic survey methods could help improve general community ecological knowledge to support conservation work and encourage long‐term, local survey efforts with actionable conservation science. For example, monitoring migratory bird collisions with buildings helps identify buildings for collision‐reduction measures and serves as a useful public engagement tool (Holpuch, 2024; Loss et al., 2019; Ocampo‐Peñuela et al., 2016). Of course, these efforts must extend beyond individual researchers and academic departments to include funding agencies and foundations if they are to create lasting change.

Whatever the solution, our results at a minimum demonstrate the importance of low‐IF journals and the science they publish in underpinning the functioning of one of the world's long‐standing conservation laws. It is important that the science they distribute be acknowledged and celebrated when considering contributions to the field and to overall conservation efforts.

## Supporting information



Supplementary Material.

Supplementary Material.

Supplementary Material.
