# Supplementary material for: Role of low‐impact‐factor journals in conservation implementation
Source: Conserv Biol. 2024 Oct 17;39(2):e14391. doi: 10.1111/cobi.14391 (PMC11959337; doi:10.1111/cobi.14391)
Supplement: Supplementary file 3 — Supplementary Material. [file COBI-39-e14391-s003.docx]

**Supplement**

**Table S1: Twenty highest Journal Impact Factors (JIF) of articles cited in listings, sorted by the average JIF for the years in which it appeared in our dataset, excluding journals that were only cited once.**

| Journal Name | Citations in Listings | Average Impact Factor |
| --- | --- | --- |
| Nature | 48 | 30.05 |
| Science | 105 | 28.43 |
| Nature Climate Change | 16 | 14.94 |
| PLoS Biology | 4 | 13.49 |
| Trends in Plant Science | 3 | 12.41 |
| Nature Geoscience | 25 | 10.78 |
| Trends in Ecology and Evolution | 28 | 10.50 |
| PNAS | 110 | 9.87 |
| Current Biology | 7 | 9.81 |
| Ecology Letters | 21 | 8.46 |
| Global Change Biology | 51 | 6.48 |
| Biological Reviews | 5 | 6.41 |
| Frontiers in Ecology and the Environment | 26 | 6.36 |
| Annual Review of Entomology | 3 | 5.75 |
| Ecological Monographs | 15 | 5.72 |
| Fish and Fisheries | 5 | 5.63 |
| Bulletin of the American Meteorological Society | 8 | 5.60 |
| Molecular Biology and Evolution | 4 | 5.56 |
| Emerging Infectious Diseases | 4 | 5.45 |
| Glob. Ecology & Biogeography | 2 | 5.27 |
